# *PPBP* and *DEFA1/DEFA3* genes in hyperlipidaemia as feasible synergistic inflammatory biomarkers for coronary heart disease

**DOI:** 10.1186/s12944-017-0471-0

**Published:** 2017-04-19

**Authors:** Yaowapa Maneerat, Kriengchai Prasongsukarn, Surachet Benjathummarak, Wilanee Dechkhajorn

**Affiliations:** 1 Department of Tropical Pathology, Faculty of Tropical Medicine, Mahidol University, Bangkok, 10400 Thailand; 2 Pramongkutklao Hospital and College of Medicine, Bangkok, 10400 Thailand; 3 Center of Excellence for Antibody Research, Faculty of Tropical Medicine, Mahidol University, Bangkok, 10400 Thailand

**Keywords:** Hyperlipidaemia, Coronary heart disease, Inflammation, Biomarker, PPBP, α-defensin

## Abstract

**Background:**

Coronary heart disease (CHD) is an important complication of atherosclerosis. Biomarkers, which associate with CHD development, are potential to predict CHD risk. To determine whether genes showing altered expression in hyperlipidaemia (H) and coronary heart disease (CHD) patients compared with controls could be CHD risk biomarkers.

**Methods:**

Control, H, and CHD groups represented atherosclerosis to CHD development. Gene profiling was investigated in peripheral blood mononuclear cells using DNA microarrays. Eight selected genes expressed only in H and CHD groups were validated by real-time quantitative reverse transcription PCR and plasma protein determination.

**Results:**

α-defensin (*DEFA1*/*DEFA3*), pro-platelet basic protein (*PPBP*), and beta and alpha2 hemoglobin mRNA expression was significantly increased in H and CHD groups compared with controls, but only plasma PPBP and α-defensin proteins were correspondingly increased.

**Conclusion:**

*PPBP* and *DEFA1*/*DEFA3* could be potential CHD biomarkers in Thai hyperlipidaemia patients.

## Background

Atherosclerosis is a complicated, progressive disease characterized by the accumulation of lipids and fibrous elements in large and medium-sized arteries. It is the major underlying cause of cardiovascular disease (CVD), which in turn is the leading cause of death in the developed world, and an important cause of morbidity worldwide. Abundant previous studies have linked dyslipidaemia to atherogenesis, and roles have been identified for inflammatory mechanisms coupled with dyslipidaemia in atheroma formation both in humans and animal models [1–3]. Early atherogenesis is characterized by leukocyte recruitment and the expression of pro-inflammatory cytokines, as shown by the fact that defective inflammatory mediators reduce atheroma formation in mice [2]. Inflammatory pathways also promote the development of thrombosis, which is a serious, late complication of atherosclerosis responsible for myocardial infarctions and coronary heart disease (CHD), associated with an increased risk of sudden death [3].

Atherogenesis and CHD involve a long preclinical process. Multiple risk factors have been identified for CHD without familial hypercholesterolaemia including behavioral, dietary, and lifestyle factors such as smoking, dietary fat intake, level of physical activity, infections (exogenous exposure), alteration of endogenous blood constituents such as lipid and lipoprotein particles, inflammation and coagulation proteins, intermediary metabolites, and oxidant markers of stress, adiposity, blood pressure, and diabetes mellitus [4]. Several clinical evaluations are available for patients with CVD and CHD, including diagnostic tests of varied accuracy, reproducibility, ease of use, and potential for patient morbidity [5].

Blood is an accessible source for diagnosing various disease processes [6], and is also an appropriate representative for atherosclerotic tissue because it contains inflammatory cells, which play an important role in atherogenesis [7]. Currently, few simple blood-based biomarkers are available for the well-defined validation of CHD patients [8]. Biomarkers such as C-reactive protein have been associated with future cardiovascular event risk [9, 10], while recent studies have revealed the potential of identifying differential gene expression in peripheral blood samples from CVD patients [11, 12]. Previous studies used the expression profiling of peripheral blood mononuclear cells (PBMCs) to study the pathogenesis, diagnosis, and pharmacokinetics of human atherosclerosis, stroke, and other vascular diseases [6, 13–15]. The present study took a similar approach to investigate gene expression differences associated with atherosclerosis and CHD complications using PBMCs from healthy controls (N), and non-familial hyperlipidaemia (H) and CHD patients. Here, we aimed to 1) investigate the intersection of gene profiling expression in H and CHD patients but not N individuals; and 2) verify whether the selected (intersected) genes could be biomarkers of CHD risk in Thai hyperlipidemia patients. Our study provides preliminary information for further in-depth studies to define appropriate biomarkers in hyperlipidaemia populations for the surveillance and prediction of long-term CHD development. In the present study, we hypothesized that our biomarkers may have valuable clinical applications. The decreased or undetectable expression of these markers in treated hyperlipidaemia patients could be used as an indicator for the effective prevention of atherogenesis and its development into CHD.

## Methods

### Materials

Dulbecco’s phosphate-buffered saline (D-PBS) and TRIzol^®^ reagent were purchased from Invitrogen (Carlsbad, CA, USA), the IsoPrep RNeasy total RNA kit was from Qiagen (Hilden, Germany), and the Affymetrix GeneChip^®^ Human Gene 1.0 ST Array was from Affymetrix (Santa Clara, CA, USA). Quantitative reverse transcription (qRT)-PCR primers were designed using Primer3 (v.0.4.0) software, GenBank sequences and based on previous studies, and were synthesized by Pacific Science Co., Ltd. (Bangkok, Thailand). The specificity of the primers for the target genes was also determined using the BLAST program [16]. Human HNP 1–3 enzyme-linked immunosorbent assay (ELISA) reagents were purchased from Hycult^®^ Biotech (Uden, the Netherlands). All other reagents were from Sigma-Aldrich (St. Louis, MO, USA).

### Study design and patient population

**Fig. 1:**
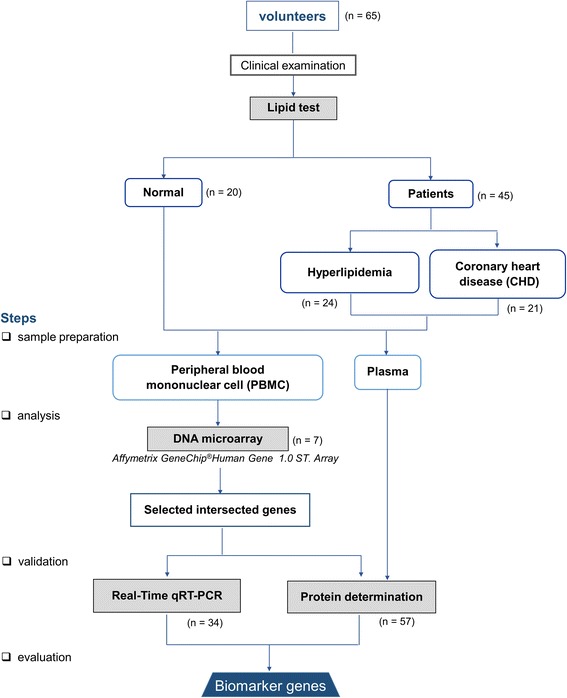
Experimental design and study population

The patient flow and experimental design are summarized in Fig. 1. The study was conducted in the Department of Tropical Medicine, Mahidol University. Approval for the study was obtained from the Ethics Committees of the Faculty of Tropical Medicine, Mahidol University (MUTM2012–031-01), and Pramongkutklao Hospital (Q004q/55_Exp). Before enrollment, all participants were informed of the study objectives, and completed an informed consent form.

#### Patients

All volunteers were unrelated males born to Thai parents. Twenty healthy controls were recruited who carried no infections, and had no underlying disease or CVD risk factors (N group). Forty-five patients were diagnosed, classified, and treated by a specialist (KP) at Pramongkutklao Hospital. They were classified into two groups based on their clinical manifestations according to the American College of Cardiology/American Heart Association criteria (2013) [17], including 24 H patients with high cholesterol levels [total cholesterol (TC), low-density lipoprotein (LDL), and high-density lipoprotein (HDL)], but with no evidence of vital organ dysfunction, and 21 patients diagnosed with CHD who were about to undergo coronary bypass grafting under the supervision of KP. No patients or controls had received any cholesterol or blood pressure-lowering medication.

### Blood sample collection and methods

Heparinized blood samples (5 ml) were collected once from healthy controls and from all patients before hyperlipidaemia treatment or coronary bypass grafting. Plasma (2 ml) was immediately collected by centrifugation of whole blood. Plasma aliquots were prepared for lipid measurement, and kept at −70 °C for the detection of plasma proteins encoded by the selected genes.

Packed blood cells were resuspended in D-PBS (Wisent Inc., Quebec, Canada) and used to isolate mononuclear cells. Approximately 2 × 10^6^ PBMCs in TRIzol (Invitrogen) were kept at −70 °C for gene expression profiling by DNA microarray analysis using Affymetrix GeneChip^®^ Human Gene 1.0 ST (Affymetrix).

### Lipid test

Lipid markers including TC, triglycerides (TG), LDL cholesterol (LDL-c), and HDL cholesterol (HDL-c) were analyzed enzymatically using kits (Randox Laboratories limited, Crumlin, UK) and a biochemistry analyzer (Architect CI 16200, Abbott Laboratories, Abbott Park, IL, USA).

### Peripheral blood mononuclear cell separation and gene expression profiling using DNA microarray analysis

PBMCs were separated by Isoprep gradient centrifugation according to the manufacturer’s recommendations (Robbins Scientific Corporation, Sunnyvale, CA, USA).

**Fig. 2:**
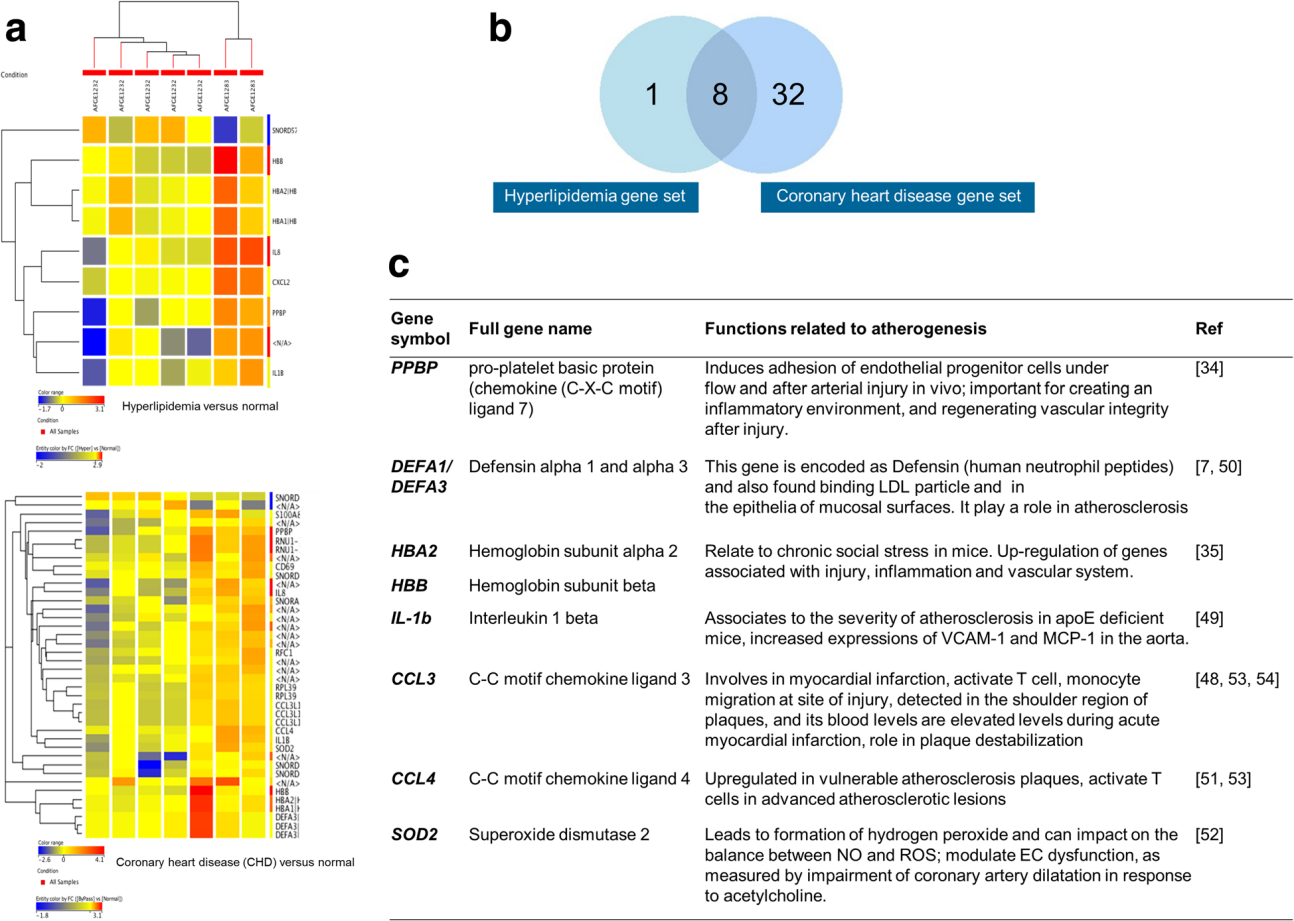
Gene expression profiling by DNA microarray. Total RNA was extracted from 2 × 10^6^ peripheral blood mononuclear cells (PBMCs) (*n* = 7). Differentially expressed genes > 2.0-fold change were further evaluated. (**a**) Heat maps of differentially expressed transcripts in PBMCs from hyperlipidaemia patients vs. control, and coronary heart disease (CHD) patients post-coronary bypass grafting vs. control. (**b**) Venn diagram illustrating the eight genes up-regulated in the two patient groups. (**c**) List of the eight genes common to both patient groups and their functions [7, 34, 35, 48–54]

Total RNA was extracted from 2 × 10^6^ PBMCs of all patients and controls (*n* = 7) using RNA isolation kits (Qiagen). Total RNA was measured using a NanoDrop ND-1000 spectrophotometer with ND-1000 3.3 software, and RNA integrity (RIN) was determined using an Agilent Bioanalyzer (Santa Clara, CA, USA). The Affymetrix GeneChip^®^ Human Gene 1.0 ST array was performed using 5 μg of total RNA with RIN ≥ 8.0, according to the manufacturer’s protocol (Affymetrix Inc). The data were analyzed by Agilent GeneSpring GX Software version 12.0. Differentially expressed genes correlating with inflammation were identified using the criteria of a > 2.0-fold increase/decrease in expression in H and CHD patients compared with the N group [18]. Figure 2 shows heat maps of differentially expressed transcripts in PBMCs from H patients vs. controls, and CHD patients post-coronary bypass grafting vs. controls. These were then further evaluated to determine their feasibility as inflammatory biomarkers of CHD development [19].

### Quantitative reverse transcription PCR analysis of the mRNA expression of selected genes

Eight intersected genes, showing increased expression in H and CHD patients, were selected for further validation. These were: α-defensin (*DEFA1/DEFA3*), pro-platelet basic protein (*PPBP*), beta haemoglobin (*HBB*), alpha 2 haemoglobin (*HBA2*), superoxide dismutase 2, (*SOD2*), chemokine ligand 3(*CCL3*), and 4 (*CCL4*), and interleukin-1β (*IL-1β)*. Table 1 lists the primers designed to amplify these genes and their expected fragment lengths. qRT-PCR was performed in duplicate [20]. Each 20-μl PCR reaction contained 10 μl of LightCycler 480 SYBR Green I Master mix (Roche Diagnostic, Mannheim, Germany) mixed with 100 ng of cDNA and 0.5 μM of each set of forward and reverse primers (Table 1). Amplification was conducted in a Bio-Rad CFX96 Real-time system (BioRad Laboratories, Inc., Hercules, CA, USA). PCR conditions were 95 °C for 5 min, followed by 45 cycles of denaturation at 95 °C for 30 s, annealing at 60 °C for 30 s, and melting curve analysis at 65 °C for 1 min [13]. ACTB primers (forward: 5′-TCACCCACACTGTGCCCATCTACGA-3′ and reverse: 5′-CAGCGGAACCGCTCATTGCCAATGG-3′) were used to normalize the relative expression level of each gene [21, 22]. 2^- (ΔΔCt)^ was used to calculate the relative expression level.

**Table 1:** Primers for gene amplification in Real-Time qRT-PCR

Gene	Accession no.	Primer sequence (5′-3′)	Length (bp)	Length (bp)	REF
*ACTB*	NM_001101.3	F: TCACCCACACTGTGCCCATCTACGA	25	295	[21, 22]
		R: CAGCGGAACCGCTCATTGCCAATGG	25		
*PPBP*	NM_002704.3	F: TTGTAGGCAGCAACTCACCC	20	135	[59]
		R: TGCAAGGCATGAAGTGGTCT	20		
*DEFA1* */DEFA3*	NM_005217.3	F: TCCTTGCTGCCATTCTCCTG	20	204	[60]
		R: TGCACGCTGGTATTCTGCAA	20		
*HBA2*	NM_000517.4	F: TCAAGCTCCTAAGCCACTGC	20	162	[61]
		R: CAGGAGGAACGGCTACCGAG	20		
*HBB*	NM_000518.4	F: GCAACCTCAAACAGACACCA	20	182	[62]
		R: CAGCATCAGGAGTGGACAGA	20		
*IL-1β*	NM_000576.2	F: CCAGCTACGAATCTCCGACC	20	180	[63]
		R: CTGCCTGCTCTTGGCTAACT	20		
*CCL3*	NM_002983.2	F: CTGCAACCAGTTCTCTGCATC	21	145	[64]
		R: TAGGAAGATGACACCGGGCT	20		
*CCL4*	NM_002984.3	F: CCGCCTGCTGCTTTTCTTAC	20	141	[65]
		R: CACTGGGATCAGCACAGACT	20		
*SOD2*	NM_001024466.1	F: TGGAAGCCATCAAACGTGACT	21	173	[66]
		R: GCCTGTTGTTCCTTGCAGTG	20		

### Determination of plasma IL-1β, PPBP, CCL3, and CCL4 protein levels

Plasma levels of IL-1β, CCL3, CCL4, and PPBP were assayed using the MILLIPLEX^®^MAP human cytokine/chemokine magnetic bead Panel III kit (Millipore Corporation, Billerica, MA, USA) according to the manufacturer’s instructions and based on a previous study [23]. Briefly, after soaking wells with 200 μl of assay buffer, 25 μl standard or assay buffer, 25 μl matrix solution or plasma (1:100), and 25 μl beads was added to the wells and incubated overnight at 4 °C with shaking. After vacuuming and washing twice with wash solution, 25 μl of detection antibodies was added and incubated for 2 h at room temperature. Then, 25 μl of streptavidin–phycoerythrin was pipetted and incubated for 30 min before vacuuming and washing, followed by the addition of 100 μl sheath fluid to each well. Measurements were performed using Luminex MAGPIX^®^ (BioRad) and interpreted by xPONENT^®^ software (Merck Millipore).

### Determination of plasma alpha (α)-defensin 1–3 levels by ELISA

Plasma α-defensin 1–3 concentrations were measured by ELISA (Hycult Biotechnology) according to the manufacturer’s instructions and as described in our previous study [13].

### Determination of plasma haemoglobin

Free haemoglobin (Hb) in plasma samples was measured spectrophotometrically (Spectrophotometer, Shimadzu UV1700, Kyoto, Japan) with Na_2_CO_3_ solution (10 mg/100 ml) as a reagent as described in a previous study [24]. Absorbance was measured at 415, 450, and 700 nm. The plasma Hb level was calculated according to the formula:

$$ \mathrm{Hb}=154.7\times \left(\mathrm{A}415\right)\hbox{-} 130.7\times \left(\mathrm{A}450\right)\hbox{-} 123.9\times \left(\mathrm{A}700\right) $$

### Statistics

Clinical data are reported as medians (upper and lower range limits). mRNA expression is represented as fold changes relative to β-actin (*ACTB*) mRNA in PBMCs. Plasma levels of PPBP, α-defensin 1–3, Hb, CCL3, CCL4, and IL -1β are non-parametric data and are also expressed as medians (upper and lower range limits). The significance of the difference between two groups was determined by the Mann–Whitney U test, and differences among the N, H, and CHD groups were determined by the Kruskal–Wallis test. mRNA expression and plasma PPBP and α-defensin 1–3 levels are represented as whisker plots, with boxes denoting the interquartile range and whiskers the minimum/maximum values. Correlations between CHD development, mRNA expression, plasma protein levels, and characteristics/clinical manifestations were determined by Spearman’s rho correlation analysis. The α level was set at <0.05 with a 95% confidence interval. All statistical analyzes were performed using SPSS version 18 software (SPSS, Chicago, IL, USA).

## Results

### Characteristics of patients and controls

General descriptions and clinical manifestations of patients and controls are compared in Table 2. The age of patients with CHD was significantly higher than that of controls and the H group (both *p* = 0.000), but there was no significant difference in age between the N and H groups. Levels of TC in the H group were significantly higher than those in N (*p* = 0.004) and CHD groups (*p* = 0.008). The LDL level in the H group tended to be higher than in the N group (*p* = 0.072) and was significantly higher than in the CHD group (*p* = 0.049). All groups showed similar TG levels (*p* > 0.05).

**Table 2:** General description and clinical manifestations of the study population

Variable	Age (year)	TC (mg/dL)	LDL (mg/dL)	TG (mg/dL)	HDL (mg/dL)
Normal	42 (23–58)	175 (156–199)	99 (60–111)	147 (70–162)	41 (31–56)
Hyperlipidaemia	42 (26–58)	223 (150–304)^b^	131 (63–190)^c^	166 (103–1181)^d^	46 (26–80)
Coronary Heart Disease (CHD)	66 (58–78)^a^	166 (115–259)	89 (44–174)	92 (72–169)	49 (37–75)

All patients and controls were male. *N* normal controls, *H and CHD* patients with hyperlipidaemia and coronary heart disease, respectively, *TC* total cholesterol, *TG* triglyceride, *HDL* high-density lipoprotein, *LDL* low-density lipoprotein

Data are shown as medians (ranges). The differences in each variable between two groups (N vs. H, H vs. CHD, and N vs. CHD) were determined using the Mann–Whitney U test. The α level was set at <0.05 at a 95% confidence interval. The significantly different variables between groups are as follows

^a^ Age of the patients with CHD was significantly more than N and H groups (*p* = 0.000)

^b^ Levels of TC in H were significantly higher than those in N (*p* = 0.004) and CHD groups (*p* = 0.008)

^c^ LDL levels in H was tended to higher than in N groups (*p* = 0.072), and significantly higher than in CHD group (*p* = 0.049)

^d^ There was significant difference in TG levels; H > CHD (*p* = 0.013); N > CHD (*p* = 0.025)

### mRNA expression in PBMC extracts

qRT-PCR findings of relative mRNA expression (mean 2-fold changes) of *PPBP*, *DEFA1/DEFA3*, *HBB*, *HBA2*, *SOD2*, *CCL3*, *CCL4*, and *IL-1β* in N, H, and CHD groups are shown in Fig. 2a. Only *PPBP, DEFA1/DEFA3*, *HBB*, and *HBA2* showed significant differences in mRNA expression in both H and CHD groups compared with the N group (Fig. 2a). The correlations between mRNA expression of the eight selected genes are summarized in Table 3.

**Table 3:** Significant correlations between mRNA expression of gene profile

Gene	*PPBP*	*DEFA1/DEFA3*	*HBA2*	*HBB*	*IL-1β*	*CCL3*	*CCL4*	*SOD2*
*PPBP*		0.3633 (*p* = 0.0410)	0.4961 (*p* = 0.0028)	0.4261 (*p* = 0.0120)			0.3533 (*p* = 0.0437)	0.3780 (*p* = 0.0301)
*DEFA1/* *DEFA3*	0.3633 (*p* = 0.0410)							
*HBA2*	0.4961 (*p* = 0.0028)			0.8487 (*p* < 0.0001)		0.4138 (*p* = 0.0167)	0.4203 (*p* = 0.0133)	0.5560 (*p* = 0.0006)
*HBB*	0.4261 (*p* = 0.0120)		0.8487 (*p* < 0.0001)			0.3626 (*p* = 0.0381)	0.3595 (*p* = 0.0368)	0.4747 (*p* = 0.0046)
*IL-1β*						0.6199 (*p* < 0.0001)	0.7173 (*p* < 0.0001)	0.5514 (*p* = 0.0007)
*CCL3*			0.4138 (*p* = 0.0167)	0.3626 (*p* = 0.0381)	0.6199 (*p* < 0.0001)		0.6269 (*p* < 0.0001)	0.5587 (*p* = 0.0006)
*CCL4*	0.3533 (*p* = 0.0437)		0.4203 (*p* = 0.0133)	0.3595 (*p* = 0.0368)	0.7173 (*p* < 0.0001)	0.6269 (*p* < 0.0001)		0.8701 (*p* < 0.0001)
*SOD2*	0.3780 (*p* = 0.0301)		0.5560 (*p* = 0.0006)	0.4747 (*p* = 0.0046)	0.5514 (*p* = 0.0007)	0.5587 (*p* = 0.0006)	0.8701 (*p* < 0.0001)	

The mRNA expressions of gene profile were non-parametric data (mean of fold change). Correlations (r_s_) between the expressions were analyzed by the Rho-Spearman correlation analysis. The level was set at <0.05 at a 95% confidence interval. Only significant correlations were shown

### Plasma levels of PPBP, α-defensin 1–3, CCL3, CCL4, and IL-1β

Plasma levels of proteins encoded by the selected genes were compared among groups using ELISA and Milliplex bead techniques (data not shown). As shown in Fig. 2b, a significant difference was only found between PPBP (*r* = 0.655, *p* = 0.034) and α-defensin 1–3 (*r* = 0.594, *p* = 0.003) levels among the three groups, which supports the *PPBP* and *DEFA1/DEFA3* expression results.

### Correlations between CHD development, mRNA expression, plasma protein levels, and clinical manifestations

**Fig. 3:**
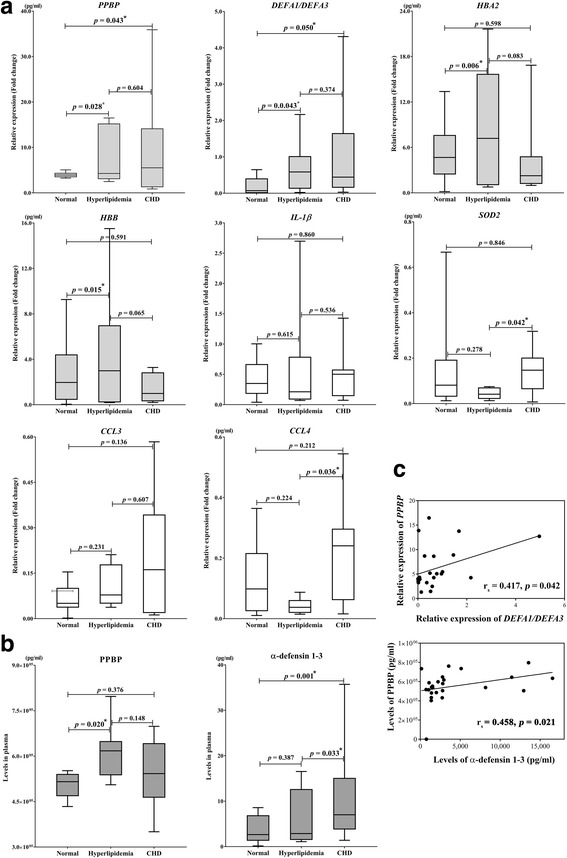
Expression of eight selected genes showing altered expression in patient groups vs. controls. (**a**) mRNA expression (2.0-fold change) relative to *β-actin* mRNA in PBMCs obtained from controls, and hyperlipidaemia and CHD patients post-bypass surgery, as determined by qRT-PCR. Data are presented as the mean 2.0-fold change relative to control ± SEM (*n* = 10). (**b**) Plasma levels (pg/ml) of PPBP and α-defensin 1–3 from healthy controls (*n* = 20), hyperlipidaemia patients (*n* = 24), and CHD patients post-bypass surgery (*n* = 21). Data are illustrated as whisker plots, with boxes denoting the interquartiles range (IQR) and whiskers the minimum/maximum values. (**c**) Correlations between relative expression of *PPBP* and *DEFA1/DEFA3,* and between plasma levels of PPBP and α-defensin 1–3. ^*^refers to a significant difference *(p > 0.05)*

All parameters in the N, H, and CHD groups were tested for correlations. Significant correlations between mRNA expression levels are summarized in Table 3, while correlations between characteristics/clinical manifestations and plasma PPBP or α-defensin 1–3 levels are shown in Table 4. Both plasma PPBP and α-defensin 1–3 levels were significantly positively correlated with age, and TC and LDL levels, but not TG or HDL levels. Additionally, significant genotype and phenotype associations between *PPBP* and *DEFA1/DEFA3* (*r*_s_ = 0.363, *p* = 0.041) and plasma PPBP and α-defensin 1–3 levels (*r*_s_ = 0.458, *p* = 0.021) were detected (Fig. 3c).

**Table 4:** Correlations between clinical data and plasma PPBP or α -defensin 1–3

Variables	Age	TC	LDL	TG	HDL
PPBP	0.604 **(** ***p*** = 0.025)*	0.577 **(** ***p*** = 0.043)*	0.543 **(** ***p*** = 0.039)*	0.081 (*p* = 0.785)	- 0.149 (*p* = 0.596)
α - de α-defensin	0.602 **(** ***p*** = 0.005)*	0.530 **(** ***p*** = 0.024)*	0.525 **(** ***p*** = 0.030)*	0.088 (*p* = 0.721)	- 0.087 (*p* = 0.714)

Plasma levels of PPBP, α-defensin 1–3, and clinical manifestations were non parametric data Correlations (r_s_) between variables were analyzed by the Rho-Spearman correlation analysis. The α level was set at <0.05 at a 95% confidence interval. * refers to a significant correlation (*p < 0.05*)

## Discussion

In this cross-sectional study, N, H, and CHD groups were representative of the long-term development of CHD, which is a common complication of atherosclerosis. This study focused on verifying markers for potential CHD prediction in non-familial Thai hyperlipidaemia patients. Our findings revealed that: 1) there was significant association between increased genotypic and phenotypic *PPBP* and *DEFA1/DEFA3* expression in H and CHD groups; and 2) *PPBP* and *DEFA1/DEFA3* expression was significantly correlation with CHD development. Based on these data, we suggest that significantly increased expression of both *PPBP* and *DEFA1/DEFA3* and their encoded proteins has the potential to be established as a synergistic predictive biomarker for CHD risk in hyperlipidaemia patients.

Previous histological studies of atherogenesis revealed that early-stage (fatty streak) atherosclerotic lesions to more complicated lesions demonstrate chronic inflammation. This develops from an interaction between plasma lipoproteins, cellular components such as monocytes, macrophages, T lymphocytes, B lymphocytes, endothelial cells (ECs), and smooth muscle cells, and the extracellular matrix of the arterial wall [2]. Polymorphonuclear neutrophils (PMNs) have also been shown to play a prominent innate inflammatory role in atherogenesis in humans [18], mice [25], and pigs [26]. PMNs were observed in plaque ruptures and erosions of human lesions, and in thrombi from acute coronary syndrome patients [27]. Previous studies have hypothesized that the number of PMNs in circulation, and the amount of PMN-produced elastase and myeloperoxidase, correlate with both atherosclerosis [27, 28] and myocardial infarction [29].

During inflammation, large amounts of intracellular proteins are released from activated PMNs into the extracellular milieu as an outcome of PMN degranulation, leakage during phagosome formation, and cell death. The amount of highly homologous human neutrophil peptides (HNPs)-1, −2, and −3, or α-defensin is more than half of the total protein content within PMN azurophilic granules [30]. α-defensin is a cysteine-rich positively-charged polypeptide produced and released from activated PMN granules. The α-defensin genes *DEFA1/DEFA3* encode HNP-1, 2, and 3 [31, 32]. Previous studies have reported that HNP 1–3 play a role in EC dysfunction during early atherogenesis. HNP levels are also markedly increased in inflammation, including sepsis and acute coronary vascular disorders [7]. Moreover, we recently showed that α-defensin 1–3 expression levels were associated with CHD development [13].

A role for platelets and platelet-derived factors in atherosclerosis has long been suggested beyond their function in the hemostatic system. Platelets are also involved in thrombus formation in response to vascular injury, and affect coronary, cerebral, and peripheral circulation [33]. Following activation, platelet α-granules rapidly release chemokines which play an important role in atherogenesis. Most chemokines attract specific leukocyte subsets to the lesion site, but they also influence the proliferation, differentiation, and degranulation of various cell types. They may exert their effects either alone or synergistically with other chemokines via different G-protein-coupled receptors expressed in target cells, some of which remain to be identified. Certain chemokines also regulate the expression or processing of the precursors of other chemokines [34].

Pro-platelet basic protein (PPBP) or chemokine (C-X-C motif) ligand 7 (CXCL7) is an encoded protein, which is a small cytokine of the CXC chemokine family. PPBP is released in large amounts from activated platelets and is involved in the response to vascular injury [35]. It stimulates various processes including mitogenesis, glucose metabolism, and the synthesis of extracellular matrix and plasminogen activator [36, 37].

In the present study, we found that the mRNA expression of *PPBP*, *DEFA1/DEFA3*, *HBA2*, and *HBB* was significantly increased in H and CHD groups of patients compared with controls, although plasma protein validation only revealed significant increases in PPBP and α-defensin 1–3. This indicates that the transcriptional and post-transcriptional processes of *PPBP* and *DEFA1/DEFA3* were successful. In agreement with a previous study [7], our observations suggest roles for platelets and neutrophils in CHD development. Because both PPBP [35] and α-defensin 1–3 [30] are released in large quantities from their respective cells, it may be feasible for them to be used as biomarkers. Table 4 summarizes the significant correlations of both plasma PPBP and α-defensin 1–3 with age, TC, and LDL, but not HDL. In support of this observation, oxidized LDL and the remnant lipoproteins beta-very low density lipoproteins have been reported to play a critical role in the pro-inflammatory reaction in atherogenesis, whereas HDL, an anti-atherogenic lipoprotein, exerts anti-inflammatory functions [1].

Hypercholesterolaemia is typically an asymptomatic condition that is often detected during routine screening. Our findings suggest the possibility of applying the plasma proteins PPBP and α-defensin 1–3 as CHD risk markers in hyperlipidaemia patients. The significant genotype (*r*_*s*_ = 0.363, *p* = 0.041) and phenotype correlations (*r*_*s*_ = 0.458, *p* = 0.021) between *PPBP* and *DEFA1/DEFA3* shown in Fig. 3c indicate that these predictive gene markers could be combined synergistically, although further studies in larger sample sizes of randomized multi-center populations should be conducted to verify this hypothesis. Previous reports of platelet–neutrophil aggregates include their detection in an acute febrile illness characterized by systemic vasculitis (Kawasaki disease), in which they were closely associated with the pathological development of coronary artery abnormalities [38]. Similarly, a platelet–neutrophil interaction was previously shown to contribute to hepatic ischemia/reperfusion injury in mice [39].

Several studies have demonstrated correlations between Hb and chronic stress in humans [40], as well as rats [40, 41], mice [35], and pigs [42]. Hb plays an important role in neuronal respiration, oxidative stress, and response to injury. Animals expressing the highest level of mRNA for Hb also showed increased expression of genes associated with the vascular system and injury response [40, 41, 43, 44]. Most studies have identified correlations between chronic social stress and brain vascular injury related to stroke, while few have investigated the relationship between Hb and CVD. For example, a recent study indicated that hemoglobin genes *HBB-B1*, *HBB-B2*, *HBA-A1*, *HBA-A2*, and *BETA-S* were potential markers of chronic social stress, which induces vascular dysfunction in mice [35]. Prior studies have also demonstrated associations between increasing levels of HbA1c and risk of death, myocardial infarction, stroke, and coronary revascularization [45–47]. In the present study, we found that increased *HBA2* and *HBB* mRNA expression was not significantly associated with translation into protein. However, we noted that *HBA2* and *HBB* mRNA expression was significantly correlated with that of *PPBP* (*r*_*s*_ = 0.4961, *p* = 0.0028; *r*_*s*_ = 0.4261, *p* = 0.012, respectively). We therefore suggest that *HBA2* and *HBB* should be investigated to determine whether they could be alternative prognostic biomarkers for CHD.

**Fig. 4:**
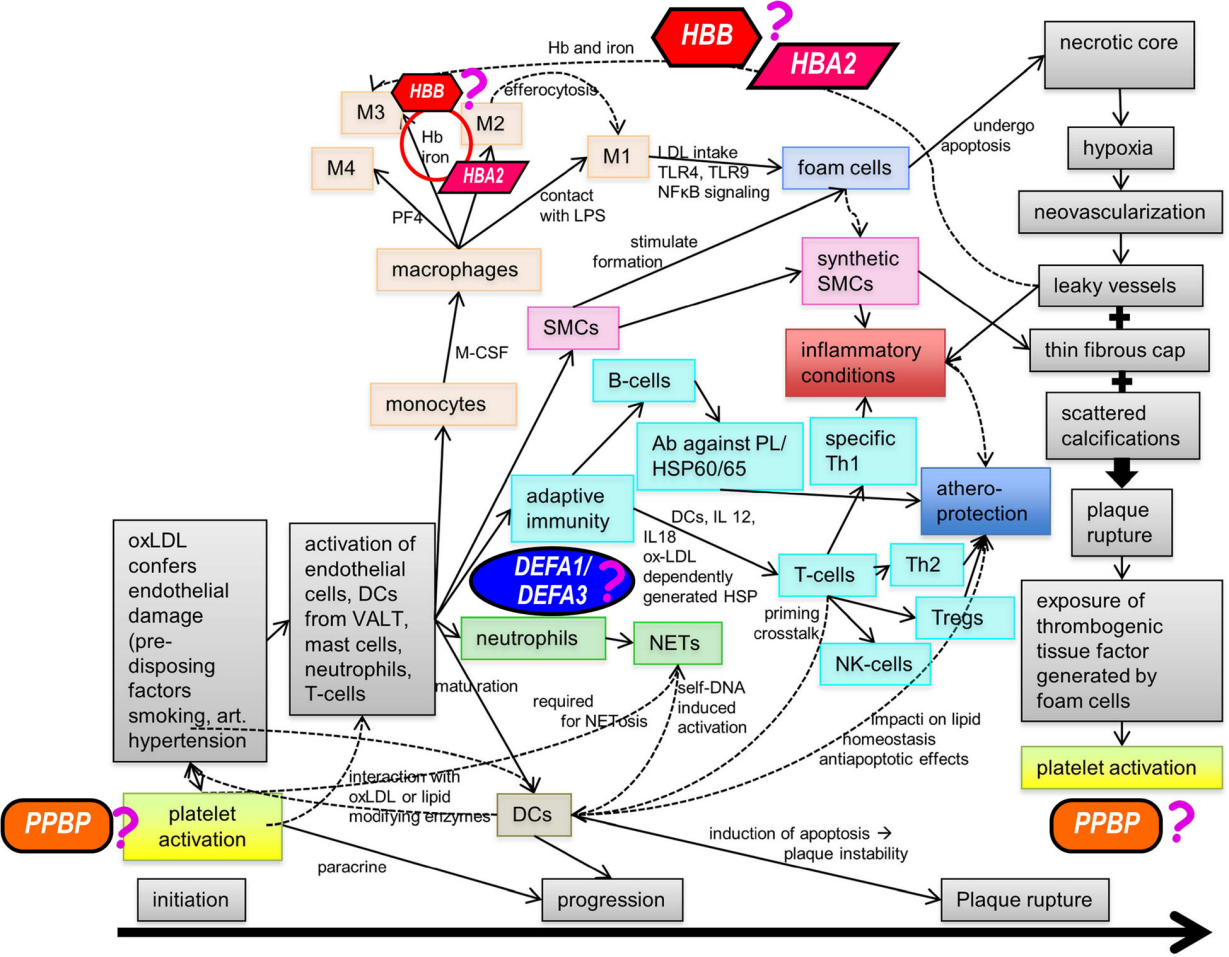
Possible roles and interplay of our potential predictive inflammatory markers; including *PPBP, DEFA1/DEFA3, HBA2*, and *HBB* in the network of atherogenesis and complications of coronary heart disease [55]

We observed no significant increase in the expression of mRNAs of the other selected genes in H and CHD groups (Fig. 3). Additionally, their plasma-encoded proteins, including IL-β, SOD2, CCL3, and CCL4, were not significantly altered. It is possible that this represents incomplete transcription or post-transcriptional processes, or that our detection techniques were not appropriate. However, we observed significant correlations among the mRNA expression levels of these genes (Table 3). These findings support the existence of interplays between pro-inflammatory cytokines, cytokines, and chemokines in CHD development. In agreement with this, previous studies suggest that our selected gene profile is involved in the development of atherogenesis and CHD [7, 34, 35, 48–54] (Fig. 2). Moreover, Fig. 4 from a previous study [55] illustrates the possible roles and network interactions of various factors in atherogenesis and CHD development. Based on our results, we suggest that our potential gene markers including *PPBP*, *DEFA1/DEFA3*, *HBA2*, and *HBB* demonstrate interplay within the network. Further investigation and confirmation of mRNA and protein expression may provide more effective predictions of CHD in hyperlipidemia patients.

Table 2 shows that the ages of the N and H groups were not significantly different. The ages of the patients with CHD were significantly higher than the N and H groups (*p* = 0.000)*.* Our result agrees with long-term atherogenesis and the development of CHD complications [2, 7]. This was the key aim of our cross- sectional study, which was designed to cover a shorter observation time, from the initiation of atherosclerosis to the development of complete CHD [56, 57].

The present study had some limitations, including 1) its small sample size and single-center analysis, 2) In determining *DEFA1/DEFA3* expression using a qRT-PCR with our designed primers, the amplicon size of the *DEFA1/DEFA3* gene (204 bp) was slightly larger than ideal (70–200 bp) for maximum efficiency [58]. Our PCR conditions were confirmed by agarose gel electrophoresis and melting curve analysis. Melting curve analysis of *DEFA1/DEFA3* showed a single peak. Similarly, agarose gel electrophoresis of the amplicon was a single band. Therefore, our *DEFA1/DEFA3* gene primer was sufficiently specific and suitable for qRT-PCR, 3) the lack of Milliplex kit optimization, which may have accounted for the observed variation in its performance; future work should determine if heparin in plasma interferes with the binding capacity, whether the 1:100 dilution of plasma samples is appropriate, the optimal incubation time, and if artifacts interfere with plasma protein levels, and 4) the lack of plasma SOD2 level determination which reflects the fact that SOD2 enzyme and cytokine/chemokine analysis could not be conducted using the same Milliplex set. An appropriate SOD2 assay should therefore be performed to verify its importance as a CHD biomarker in future studies.

## Conclusion

We conducted a cross-sectional study of the evaluation of CHD predictive biomarkers in non-familial hyperlipidaemia. Among eight potential markers, *PPBB* and *DEFA1/DEFA3* were the most strongly correlated with CHD development, and show promise for further application as inflammatory markers to synergistically predict the risk of CHD development in Thai hyperlipidaemia patients.

## Abbreviations

CHD: Coronary heart disease
ELISA: Enzyme-linked immunosorbent assay
HDL: High-density lipoprotein
HNP 1–3: Human neutrophil peptides 1–3
LDL: Low-density lipoprotein
PBMC: Peripheral blood mononuclear cells
qRT-PCR: Quantitative reverse transcription-polymerase chain reaction
TC: Total cholesterol
TG: Triglyceride
α-defensin: Alpha-defensin
